# Ultrastructural and Immunohistochemical Study of Double and Combined Intravitreal Administration of Antifungal Agents in the Retina of New Zealand Albino Rabbits: An Experimental Protocol

**DOI:** 10.3390/jof11080564

**Published:** 2025-07-29

**Authors:** Sofia Karachrysafi, Maria Kourti, Sophia Tsokkou, Despoina Ioannou, Evangelia Kofidou, Georgios Delis, Sotiris Sotiriou, Athanasios Karamitsos, Maria Xioteli, Ioanna Dori, Penelope Anastasiadou, Ioannis Konstantinidis, Dimitrios Kavvadas, Fotios Chatzinikolaou, Anastasia Komnenou, Vasileios Karampatakis, Antonia Sioga, Theodora Papamitsou

**Affiliations:** 1 Research Team “Histologistas”, Interinstitutional Postgraduate Program “Health and Environmental Factors”, Department of Medicine, Faculty of Health Sciences, Aristotle University of Thessaloniki, 54124 Thessaloniki, Greece; stsokkou@auth.gr (S.T.); dioana@auth.gr (D.I.); sotiris_sot@hotmail.com (S.S.); thanos_karamitsos@yahoo.gr (A.K.); panastasiadoy@yahoo.gr (P.A.); ikonsc@auth.gr (I.K.); kavvadas@auth.gr (D.K.); sioga@auth.gr (A.S.); thpapami@auth.gr (T.P.); 2 Laboratory of Histology-Embryology, School of Medicine, Faculty of Health Sciences, Aristotle University of Thessaloniki, 54124 Thessaloniki, Greece; 3 2nd Department of Pediatrics, AHEPA Hospital, Department of Medicine, Faculty of Health Sciences, Aristotle University of Thessaloniki, 54124 Thessaloniki, Greece; 4 School of Veterinary Medicine, Faculty of Health Sciences, Aristotle University of Thessaloniki, 54124 Thessaloniki, Greece; evikofidou@gmail.com (E.K.); natakomn@gmail.com (A.K.); 5 Laboratory of Pharmacology, School of Veterinary Medicine, Faculty of Health Sciences, Aristotle University of Thessaloniki, 54124 Thessaloniki, Greece; delis@vet.auth.gr; 6 Department of Pathology, AHEPA Hospital, Faculty of Health Sciences, Aristotle University of Thessaloniki, 54124 Thessaloniki, Greece; 7 Laboratory of Anatomy, Histology and Embryology, School of Veterinary Medicine, Faculty of Health Sciences, Aristotle University of Thessaloniki, 54124 Thessaloniki, Greece; mchiotel@vet.auth.gr (M.X.); idori@vet.auth.gr (I.D.); 8 Department of Oral Medicine/Pathology, School of Dentistry, Faculty of Health Sciences, Aristotle University of Thessaloniki, 54124 Thessaloniki, Greece; 9 Laboratory of Forensic Medicine and Toxicology, School of Medicine, Faculty of Health Sciences, Aristotle University of Thessaloniki, 54124 Thessaloniki, Greece; fotischatzin@auth.gr; 10 Laboratory of Experimental Ophthalmology, School of Medicine, Faculty of Health Sciences, Aristotle University of Thessaloniki, 54124 Thessaloniki, Greece; karophth@gmail.com

**Keywords:** voriconazole, micafungin, retina, IL-6, TNF-a, immunohistochemistry, optical microscope, electron microscope, ultrastructural, histological

## Abstract

Introduction: Fungal endophthalmitis (FE) is a rare but serious intraocular inflammatory disorder, resulting from an infection of the vitreous cavity from either endogenous or exogenous components that ultimately results in blindness. This current research study aims to elucidate the histological effects of the intravitreal injection of the maximum safe dosage of voriconazole and micafungin on the retina and investigate potential histological alterations after the double and combined administration of voriconazole and micafungin. Methodology: Nine New Zealand Albino Rabbits were randomly assigned into three groups (V2, M2, and VM), and in each, voriconazole, micafungin, and a combination of the two medications were administered respectively. After the administration of the antifungal agents, the animals were sacrificed and their retinas were retrieved and studied under optical and electron microscopes. The immunohistochemical markers TNF-a and IL6 were also studied. Results: TNF-a was positive in the VM group, as it was found to be mildly positive in the presence of apoptotic cells in the ganglion cell layer. Conclusions: This study revealed voriconazole has a greater toxicity in a multi-dosage administration. However, micafungin revealed a greater toxicity than voriconazole from the extent of the lesions observed.

## 1. Introduction

Fungal endophthalmitis (FE) is considered a rare but serious intraocular inflammatory disorder, resulting from an infection of the vitreous cavity from either endogenous or exogenous components that ultimately results in blindness [1]. Exogenous components include penetrating trauma and surgeries, whereas endogenous components include hematogenous spread of the infection [2]—which results in slow vision loss due to choroiditis or retinitis that is able to spread to the vitreous body [3]. The Centers of Disease Control and Prevention (CDC) state that exogenous endophthalmitis is more common than endogenous, with an estimated case reaching only 2 to 15% regarding endogenous endophthalmitis [4].

### 1.1. Exogenous Endophthalmitis

Exogenous endophthalmitis is unilateral intraocular inflammation caused by the entrance of fungal pathogens in the eye via direct inoculation [5], including post-surgery, penetrating trauma, and after advanced infectious keratitis [5,6]. It is stated as either acute or chronic based on the duration of the infection, with an onset greater than 6 weeks being classified as chronic. Exogenous endophthalmitis can be both fungal and bacterial, but with the *Candida* species being the most prevalent causative organism [5].

### 1.2. Endogenous Endophthalmitis

Although less common, endogenous endophthalmitis is more aggressive and even leads to vision-threatening sequelae [7]. In most cases, endogenous endophthalmitis appears bilaterally [8]. Both bacterial and fungal cases were recorded in the same percentage with bacterial cases of *Streptococcus pneumoniae*—a gram-positive lancet—shaped bacterium [9,10] and *Staphylococcus aureus*—a gram-positive bacterium [11]—being the most prevalent bacterial types in both North America and Europe [8]. In East Asia, on the other hand, *Klebsiella pneumoniae*—a gram-negative, encapsulated, non-motile bacterium—is mainly responsible for most cases [8,12].

Additionally, *Candida albicans* is the most common cause of fungal infections [8]—an endogenous secondary candidemia that can also be caused by exogenous traumatic incidents [13]—followed by aspergillus as the second most common cause.

### 1.3. Etiology and Epidemiology

The retrospective study of Das T. et al. [14], which was conducted in India, revealed that, of patients suffering FE, 46.8% of the cases were postoperative, 35.6% were posttraumatic, and 17.5% were endogenous cases [14]. It was stated that 39% of the cases recorded were due to *Aspergillus* species infections, 15.1% were due to *Candida* species exposure, 15.9% were due to *Fusarium* species exposure, and 30% were due to other fungal infections [14].

The study of Wan L. et al. stated that 9.4% of fungal keratitis patients developed endophthalmitis [15], with risk factors being the application of topical steroid use, past corneal laceration suturing, and a large corneal ulcer size reaching 10 mm diameter and greater, as well as hypopyon and aphakia. The most frequent type of fungi reported in this study were *Fusarium*, with a percentage reaching 40.5%, and *Aspergillus*, with 16.2% [15].

### 1.4. Therapeutic Approaches

The early diagnosis and initiation of systemic and intravitreal therapy are important to reduce both mortality and ocular morbidity. Close follow-up with examinations at least twice weekly initially is necessary to assess the response to therapy. Despite the emergence of new drugs, treatment remains challenging in many cases. In comparison with antibacterial drugs, antifungal medications have shown to have lower efficacy due to their mechanical action—antifungal agents have dose-dependent fungicidal action and lower tissue penetration [16]. Polyene antifungals (such as amphotericin B and natamycin) and azole derivatives (including ketoconazole, voriconazole, itraconazole, and fluconazole) are commonly used to treat fungal infections. These medications can be administered systemically—either orally or intravenously—as well as topically or directly into the eye to provide a more localized therapeutic effect. In recent years, echinocandins (caspofungin, micafungin, and anidulafungin) have gained prominence as well. Primarily used systemically, echinocandins are effective against systemic fungal infections, particularly those caused by *Candida* and *Aspergillus* species. Their growing role in treatment is supported by their low toxicity, comparable efficacy to established drugs like amphotericin B and fluconazole, and favorable cost-effectiveness. Given these advantages, echinocandins represent a promising development in the management of fungal endophthalmitis, highlighting the need for further research into their therapeutic potential in this context [16,17].

#### 1.4.1. Amphotericin B

One of the main therapeutic approaches used for FE is intravitreal amphotericin (AMB)—a polyene that binds to sterols surfaces in the fungal cell membrane with the aim of altering its permeability by opening pores that will cause the leakage of the intracellular material and lead fungal cell death [18,19,20,21]. AMB has shown potent antifungal activity clinically against the fungal species *Candida*, *Aspergillus*, and *Fusarium* [22]. Therefore, it is used as a therapeutic agent in the treatment of ocular fungal infections. It has demonstrated therapeutic efficacy in fungal keratitis when administered subconjunctivally, topically, in the intraarterial chamber, intravitreally, and intrastromally [23].

#### 1.4.2. Natamycin

Natamycin is currently the only antifungal agent commercially available for the treatment of fungal keratitis, conjunctivitis, and blepharitis. It is a member of the polyene class and functions by specifically binding to ergosterol, a vital component of the fungal cell membrane responsible for maintaining its structural integrity. Unlike amphotericin B, natamycin does not increase membrane permeability or cause leakage of intracellular contents; instead, its fungicidal effect is solely mediated through ergosterol binding [24]. It is commonly used as a first-line treatment for fungal keratitis, primarily because of its effectiveness against *Aspergillus* and *Fusarium* species—key pathogens in ocular fungal infections—and its superior ocular tolerability compared to other antifungal agents.

#### 1.4.3. Voriconazole

Voriconazole is an azole antifungal used as a new medical approach to FE. It is a second-generation triazole that acts on the enzyme cytochrome P450 (CYP) and thus prevents the ergosterol synthesis in the cytoplasmic membrane, inhibiting fungal growth. It can be administered orally, intravenously, and intravitreally [18,25]. Research suggests that it has strong biological activity against *Candida*, *Aspergillus*, *Fusarium*, and filamentous fungal species [25,26,27,28]. Voriconazole is more favorable in comparison with AMB due to the lower risk in association with retinal toxicity and its wider spectrum. Thus, is the first choice based on the data up to now [18]. On the contradictory point, as voriconazole is metabolized in the liver it can cause hepatotoxicity. Additionally, it can cause embryotoxicity, leading to teratogenicity, skin rashes, and reversible vision disorders [18,19,27,28,29,30,31].

#### 1.4.4. Micafungin

Micafungin (Mycamine, FK463) is a water-soluble, semisynthetic lipopeptide synthesized by the chemical modification of a fermentation product from *Coleophoma empetri* [32]. It inhibits, like all echinocandins as mentioned above, selectively the synthesis of 1,3-beta-D glucan, an essential component of the fungal cell wall. The continuous synthesis of 1,3-β-D-glucan is vital for maintaining the integrity of the fungal cell wall, and inhibition leads to osmotic instability and ultimately cell lysis. Fungicidal activity is observed in the majority of *Candida* species, with low in vitro minimum inhibitory concentrations (MICs) for *C. albicans*, *C. glabrata*, and *C. tropicalis* and relatively higher MICs for *C. krusei* and *C. parapsilosis* [33]. The fungistatic activity was observed in *Aspergillus* species, where echinocandins in particular show effects on the active cell growth of hyphae, leading to damage to these structures [34]. Micafungin is not active against *Cryptococcus neoformans*, even though its cell wall contains 1,3-beta-D-glucan, and also shows little activity against *Fusarium* spp. and zygomycetes [35].

### 1.5. Immunohistochemical Markers

#### 1.5.1. Interleukin 6—IL-6

Interleukin 6 (IL-6)is a cytokine produced by various cells, including T- and B-lymphocytes, monocytes/macrophages, neutrophils, fibroblasts, endothelial cells, hepatocytes, and mesangial cells, as well as cancer cells [36,37]. IL-6 has a dual effect on the eyes. Firstly, it protects ocular tissues from harmful infections and additionally it can damage sensitive ocular elements through undesirable neovascularization or recrudescence of inflammation [38].

#### 1.5.2. Tumor Necrosis Factor-Alpha—TNF-a

TNF-a is an important pro-inflammatory cytokine with pleiotropic functions that is composed primarily of T-lymphocytes and macrophages/monocytes and to a lesser extent of neutrophils and mast cells [39,40]. During periods of acute inflammation, TNF-a leads to necrosis or apoptosis. It plays an important role in the resistance to infection and cancer [39,41,42,43,44,45,46,47]. TNF-a is involved in the pathogenesis of many ocular inflammatory diseases [39].

### 1.6. Aim of Study

The current research aimed to elucidate the histological effects of the intravitreal injection of the maximum safe dosage of voriconazole and micafungin according to the available literature on the retina and investigate the potential histological alterations after the double and combined administration of voriconazole and micafungin. To our knowledge, there are not up to this day similar histological and immunohistochemical studies of the impact of the stated drugs after intravitreal injection.

## 2. Methodology

### 2.1. Materials

This study was conducted using the species of New Zealand Albino Rabbits of the sexes of male and female. In total, nine (9) New Zealand White Rabbits were used at the age of 5 months [18]. The choice of age and species of used was based on previous studies found from the literature investigation due to their ocular similarity to humans and manageable eye size. they are more commonly used in ophthalmological experimental protocols as they are easier to manage to obtain tissues for processing under optical and electron microscopes. There are also studies using rats or pigs, but rats have very small eyes, and obtaining sufficient tissue from the retina for treatment for the optical microscope would not be possible. Larger species or rats were also avoided due to practicality and tissue yield [36,37,38,48,49].

### 2.2. Replacement, Reduction, Refinement (3Rs) Condition

The selection of the species and number of experimental animals was made by applying the 3Rs condition (Replacement, Reduction, Refinement) to ensure the use of the smallest possible number of laboratory animals as well as the least possible suffering of the animals without simultaneously affecting the research results. Thus, the right eye of the animals was the intervention group while the left eye constituted the control group, reducing the number of animals required for the protocol because no additional animals were required as a control group.

### 2.3. Randomization

The rabbits were randomly assigned into, 3 groups; double administration of voricon-azole (V2), double administration of micafungin (M2), and a combination of the two medications (VM). The randomization was conducted using the Animal Randomization Tool (ACME, https://acmeresearchlabs.in/animal-randomization-tool/, accessed on 12 February 2019) [50]. Each rabbit was assigned a number from 1 to 15, and using the randomization tool a total of 3 groups were constructed, with each group consisting of 3 rabbits. As stated above, the left eye from each group was the control group (C2). Thus, it is considered as a supplementary group rather than an additional, one since the eyes used were from the already assigned rabbits (Table 1).

### 2.4. Medication and Dosages of Administration

Injections were given on days 0 and 4, with euthanasia on day 14, i.e., 10 days after the completion of intravitreal infusions. An ophthalmological examination was carried out before sacrificing the animals. In groups V2 and VM, voriconazole solution was administered at a dose of 40 μg/0.1 mL to achieve an intravitreal concentration of 25 μg/mL [48,49]. The calculation was made assuming that the average volume of the vitreous body of a rabbit is approximately 1.6 mL, as the literature shows a variation [48,50,51,52,53,54], while in groups M2 and VM micafungin solution was administered at a dose of 25 μg/0.1 mL [13]. Group C2 was administered 0.1 mL BSS solution. The substances were administered by intravitreal infusion.

### 2.5. Housing and Care of Animals

The experimental animals were housed in a specifically designed area where there was an air conduction system to maintain ventilation, with constant temperature and regulated lighting. The cages of the experimental animals were made with materials that do not harm the health of the animals and with provision to achieve their daily cleaning easily and without the animals coming into contact with dirt, maintaining all hygienic criteria. There was always fresh clean water, concentrated food, and grass at their disposal, which were renewed daily.

### 2.6. Description of Equipment

For the surgical procedure, the “Companion Animal Clinic”, an ophthalmological surgery clinic, was used, as it meets the terms and conditions of a fully equipped and aseptic surgery where a surgical microscope and all the necessary equipment are present. The daily eye examination was performed using a portable slit lamp.

### 2.7. Description of the Procedure

**Figure 1:**
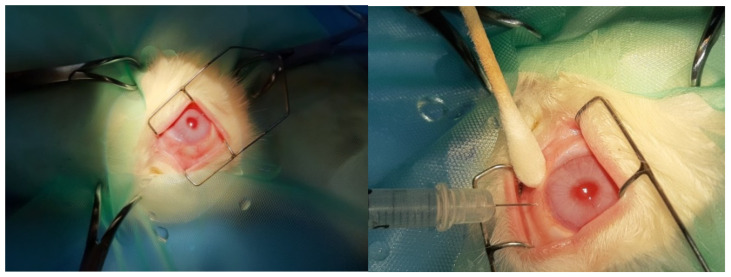
Intravitreal injection procedure into rabbits’ eyes during the research protocol.

**Figure 2:**
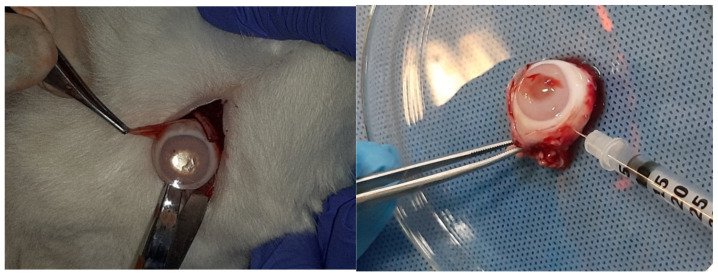
Surgical extraction of rabbit eyes (**left**) and initiation of fixation with intravitreal glutaraldehyde injection into the eyes (**right**).

The rabbits were weighed and anesthetized with dexmedetomidine 50 μg/kg, butor-phanol 0.1 mg/kg (im) for premedication and ketamine 25 mg/kg im and propofol 0.5 mg/kg iv (where a longer time was required for anesthesia). The surface of the eyes was anesthetized with 0.5% proparacaine hydrochloride. Pupils were dilated with tropi-camide 1%. Voriconazole, micafungin, and BSS solutions were administered to the center of the vitreous body using a 30-gauge needle. A cotton swab was placed over the injection site for 30 s to reduce the risk of leakage (Figure 1). Before the sacrifice, indirect ophthalmoscopy and slit lamp use were performed. The euthanasia of the animals was performed by administration of dexmedetomidine, ketamine, propofol, and KCl. This was followed by the eyes’ removal and sample extraction from the retinas so that they were properly processed for observation under the optical microscope and for immunohistochemical study, as well as under the electron microscope. Animals in all groups were sacrificed 10 days after the end of the intravitreal injections. With eye extraction, intravitreal glutaraldehyde was injected for initial fixation (Figure 2). Tissue samples taken during the experimental protocol were initially observed macroscopically.

### 2.8. Preparation for Observation Under the Optical Microscope

The retinal samples were cut into blocks 0.5 cm to 4 cm thick, placed in special capsules, and immediately immersed in a 10% formaldehyde fixative solution of 35% formaldehyde. The samples underwent dehydration and clarification for paraffin embedding. Dehydration was done in an ascending alcohol series for six hours (76%, 96%, 100%, and 100%), followed by a 4 h clarification in xylol. Capsules were then soaked in liquid paraffin for four hours. Tissues were placed in special metal molds, with liquid paraffin and cooled at 4 degrees Celsius for 20 min. Each paraffin cube was sectioned with a semi-automatic microtome (Leica RM2255, Leica, Wetzlar, Germany) at 3 μm, producing 10 incisions. From these sections, the first three were mounted on slides, while the remaining seven were placed on positively charged slides and dried at room temperature for one hour. The first 3 incisions were placed in a furnace for one hour at 65 degrees Celsius, deparaffinized in xylol for 10 min, and then hydrated in descending ethanol for twenty minutes (100%, 100%, 96%, and 76%). They were stained with hematoxylin for five minutes, rinsed in tap water for another five minutes, briefly differentiated with 1% solution for one second, stained with eosin for one minute, dehydrated for five minutes in ethanol, and placed for another five minutes in xylol for clarification. Finally, the slides were covered with “balm of Canada” for microscopy. The remaining positively charged slides were used for immunohistochemistry methods with IL-6 (Santa Cruz, CA, USA, solution 1:100) and TNF-a (Abcam (Cambridge, UK), solution 1:100). Sections were incubated in a humidified chamber with 200 μL IL-6 antibody with a dilution of 1:100 for 1 h or TNF-a with a dilution of 1:100 for 30 min. Then, the samples were rinsed for 5 min with wash buffer, incubated with Peroxidase blocking solution for 10 min, rinsed again for 5 min, and incubated with Dako Polymer EnVision HRP (Agilent Technologies, Santa Clara, CA, USA) for 25 min at room temperature. Proceeding, the samples were rinsed with wash buffer for 5 min and 3–4 drops of ready-made fresh DAB chromogen (Sigma-Aldrich, St. Louis, MO, USA) were added to the sample and were then incubated for 10 min at room temperature. Moreover, they were rinsed with distilled water and wash buffer for 5 min, respectively. Additionally, 3–4 drops of hematoxylin were added to the sample and incubated for 2 min at room temperature. Again, wash buffer and distilled water were used for rinsing for 5 min. Finally, dehydration was performed in an ascending series of alcohols, immersion in xylol, and coverage with a coating agent. The sample was observed for diagnosis under the optical microscope. Therefore, eosin–hematoxylin staining and immunohistochemical staining for immunohistochemical markers IL-6 and TNF-a were performed for each subgroup.

Given that IL-6 has a dual effect on the eyes, protecting ocular tissues from harmful infections and damaging sensitive ocular elements through undesirable neovascularization or recrudescence of inflammation [38], IL-6 was considered an important inflammatory biomarker to be studied in the present study. TNF-a is also involved in the pathogenesis of many ocular inflammatory diseases [39]. In this context, it seemed necessarily important to use it to check voriconazole’s and micafungin’s ocular action and possible side effects, particularly on retinal tissues.

### 2.9. Double Blind Assessment

All slides were examined under the optical microscope by 2 independent professional observers. Observers were blinded to the rabbit ID and group assignments to ensure objective evaluation. To eliminate any bias during the evaluation process, assessments were conducted separately and recorded electronically. Immunohistochemical staining intensity was assessed using a scale of crosses: negative (no cross, −), mild (one cross, +), moderate (two crosses, ++), and intense (three crosses, +++).

### 2.10. Preparation for Observation Under the Electron Microscope

Retinal tissue samples were dissected into pieces smaller than <1 cm^3^, fixed in 3% glutaraldehyde for 2 h, rinsed in phosphate buffer for 10′, and post-fixed in 1% osmium tetroxide (OsO4) for 1 1/2 h. Samples were washed with phosphate buffer for 10′ and twice distilled for 10′. Staining and simultaneous fixation were done with uranyl acetate 1% for 16 h and then dehydration with an ascending series of alcohols (30° for 5′, 50° for 5′, 70° for 5′, 96° for 5′, and 100° for 5′ × 6 times). Samples were encapsulated in Epon resin and very thin incisions (60–90 nm) were taken. Finally, sections were treated with Reynold’s stain and observed using a TEM JEOL 1011 electron microscope at 80 kV (JEOL-Tokyo, Tokyo, Japan).

### 2.11. Ethics Committee Approval

This study was approved by the Veterinary Directorate of PKM, Department of Animal Health & Veterinary Perception, Medicines and Applications (YZ-KAFE), under protocol number 57253 (211), dated 15 February 2019, and by the “Bioethics and Ethics Committee” of the Medical Department of the School of Health Sciences of the Aristotle University of Thessaloniki under approval number 4.209, dated 17 July 2019.

## 3. Results

### 3.1. Results of Optical Microscope—Eosin–Hematoxylin Staining and Immunohistochemical Staining for IL-6 and TNF-a Markers

#### 3.1.1. Group C2

**Figure 3:**
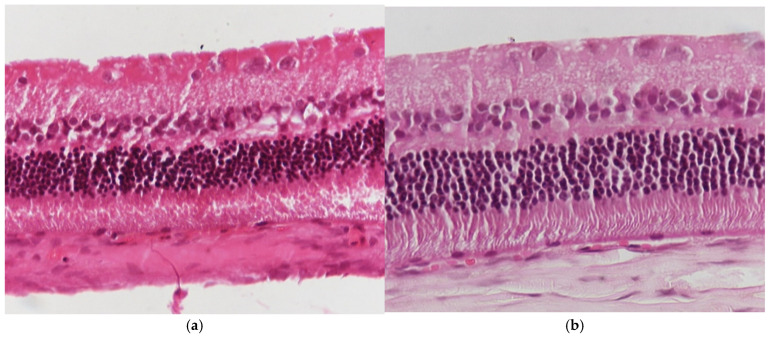
(**a**) Group C2. Retina with absence of morphological lesions H&E. ×160. (**b**) Group C2. Retina with absence of morphological lesions H&E. ×200.

**Figure 4:**
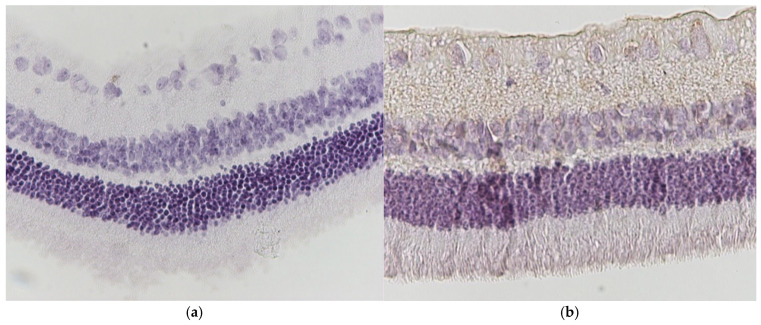
(**a**) Group C2. Retina with negative (−) IL-6 staining. ×160. (**b**) Group C2. Retina with negative (−) IL-6 staining. ×200.

**Figure 5:**
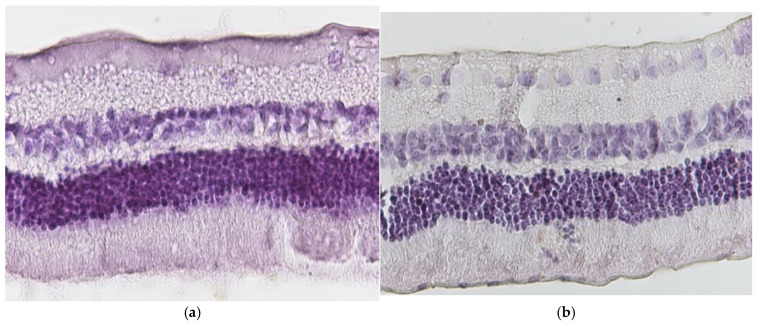
(**a**) Group C2. Retina with negative (−) TNF-a staining. ×200. (**b**) Group C2. Retina with negative (−) TNF-a staining. ×200.

Regarding the results of Group C2, the application of hematoxylin–eosin (H&E) staining showed no morphological alterations in the retina (Figure 3a,b), while immunohistochemical stains for IL-6 (Figure 4a,b) and TNF-a markers (Figure 5a,b) were negative (−) in all samples.

#### 3.1.2. Group V2

**Figure 6:**
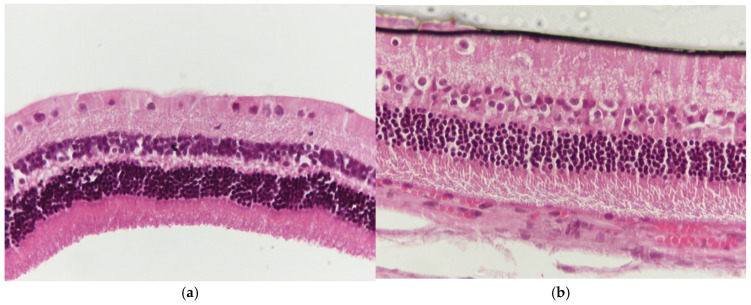
(**a**) Group V2. Retina with absence of morphological lesions. H&E. ×160. (**b**) Group V2. Retina with absence of morphological lesions. H&E. ×200.

**Figure 7:**
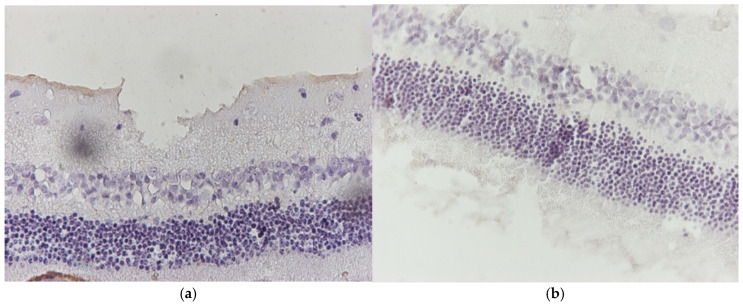
(**a**) Group V2. Retina with negative (−) IL-6 staining. ×160. (**b**) Group V2. Retina with negative (−) IL-6 staining. ×200.

**Figure 8:**
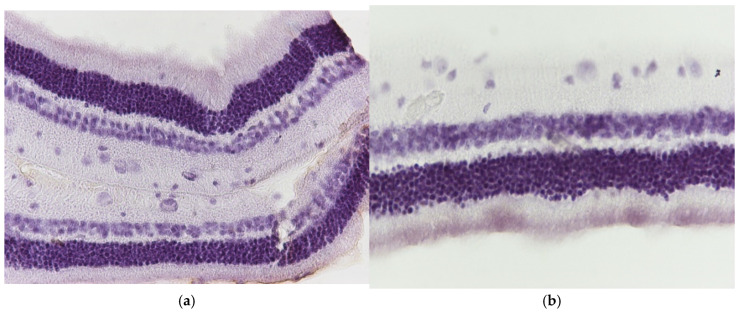
(**a**) Group V2. Retina with negative (−) TNF-a staining. ×160. (**b**) Group V2. Retina with negative (−) TNF-a staining. ×200.

Regarding the results of group V2, the application of eosin–hematoxylin staining showed no morphological alterations in the retina (Figure 6a,b), while immunohistochemical stains for IL-6 (Figure 7a,b) and TNF-a (Figure 8a,b) were negative in all samples.

#### 3.1.3. Group M2

**Figure 9:**
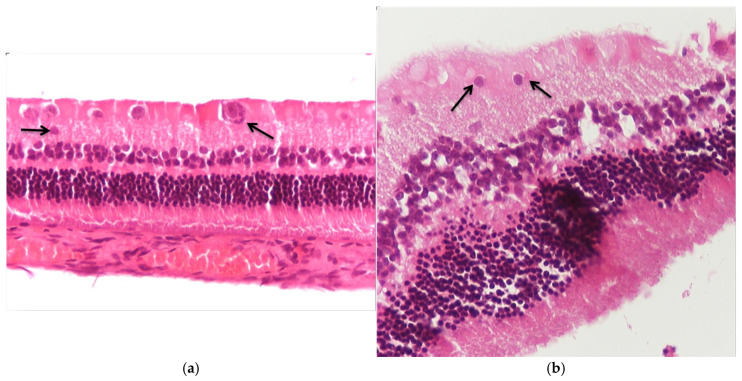
(**a**) Group M2. Shrinkage of cytoplasm in places in the ganglion cell layer (🡺). H&E. ×160. (**b**) Group M2. Shrinkage of cytoplasm in places in the ganglion cell layer (🡺). H&E. ×200.

**Figure 10:**
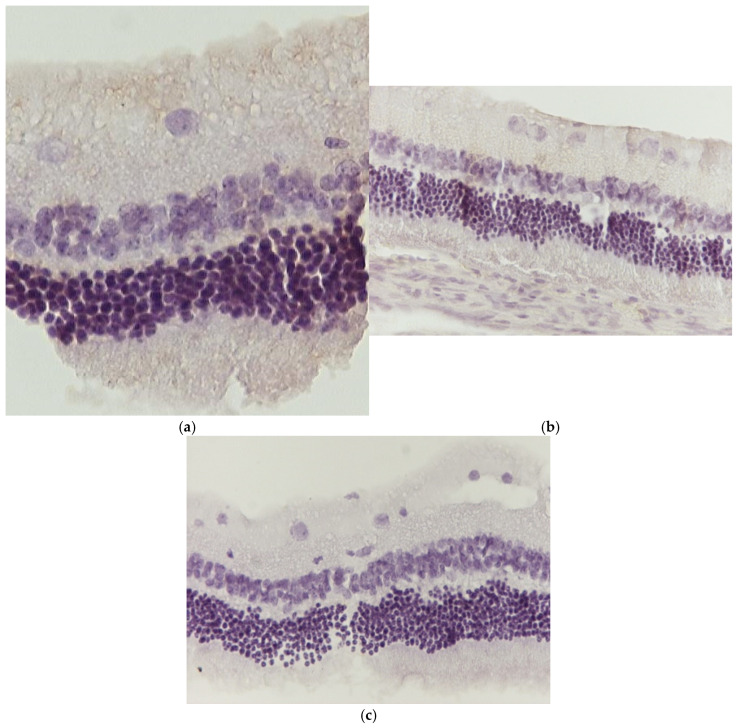
(**a**) Group M2. Retina with negative (−) IL-6 staining. ×200. (**b**) Group M2. Retina with negative (−) TNF-a staining. ×160. (**c**) Group M2. Retina with negative (−) TNF-a staining. ×160.

Regarding the results of group M2, the application of eosin–hematoxylin staining revealed shrinkage of ganglion cell cytoplasm in the ganglion cell layer of the particular retina (Figure 9a,b), while immunohistochemical stains for IL-6 (Figure 10a) and TNF-a (Figure 10b,c) were negative in all samples.

#### 3.1.4. Group VM

**Figure 11:**
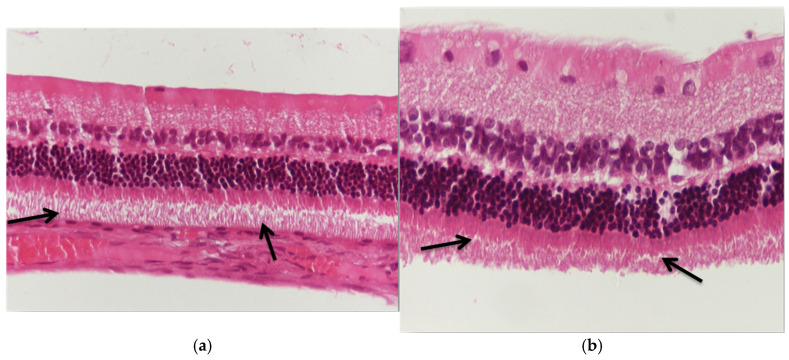
(**a**) Group VM. Thinning in the outer section of the rods and cones (🡺) H&E. ×160. (**b**) Group VM. Thinning in the outer section of the rods and cones (🡺) H&E. ×200.

**Figure 12:**
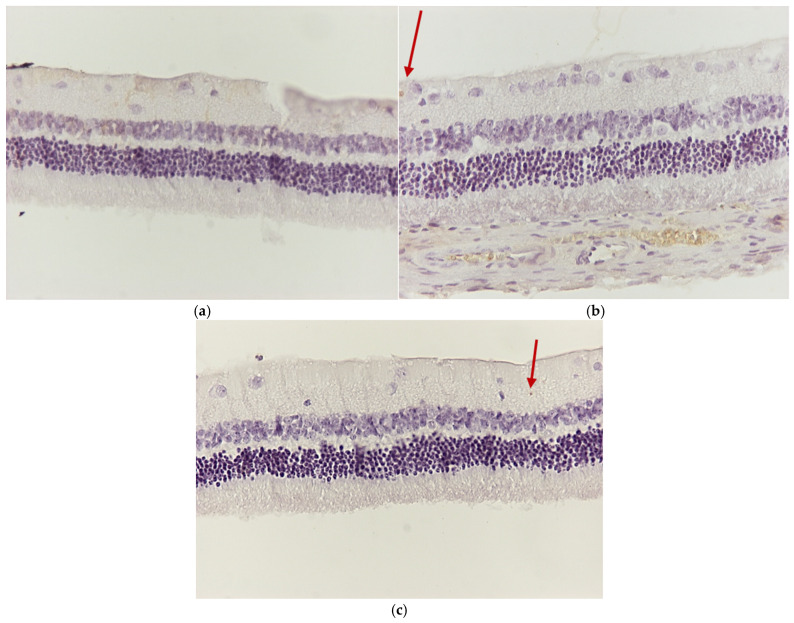
(**a**) Group VM. Retina with negative (−) IL-6 staining. ×160. (**b**) Group VM. Retina mild positive (+) staining in the presence of one apoptotic cell (🡺). TNF-a. ×160. (**c**) Group VM. Retina mild positive (+) staining in the presence of one apoptotic cell (🡺). TNF-a. ×160.

Regarding the results of the VM group, the application of eosin–hematoxylin staining revealed thinning in the outer part of the rods and cones in the retina (Figure 11a,b). The immunohistochemical staining for IL-6 (Figure 12a) was detected as negative in all samples, while that for TNF-a was found to be mildly positive with the presence of apoptotic cells in the ganglion cell layer in two of the three rabbit eyes of the group (Figure 12b,c).

### 3.2. Electron Microscopy

#### 3.2.1. Group C2

**Figure 13:**
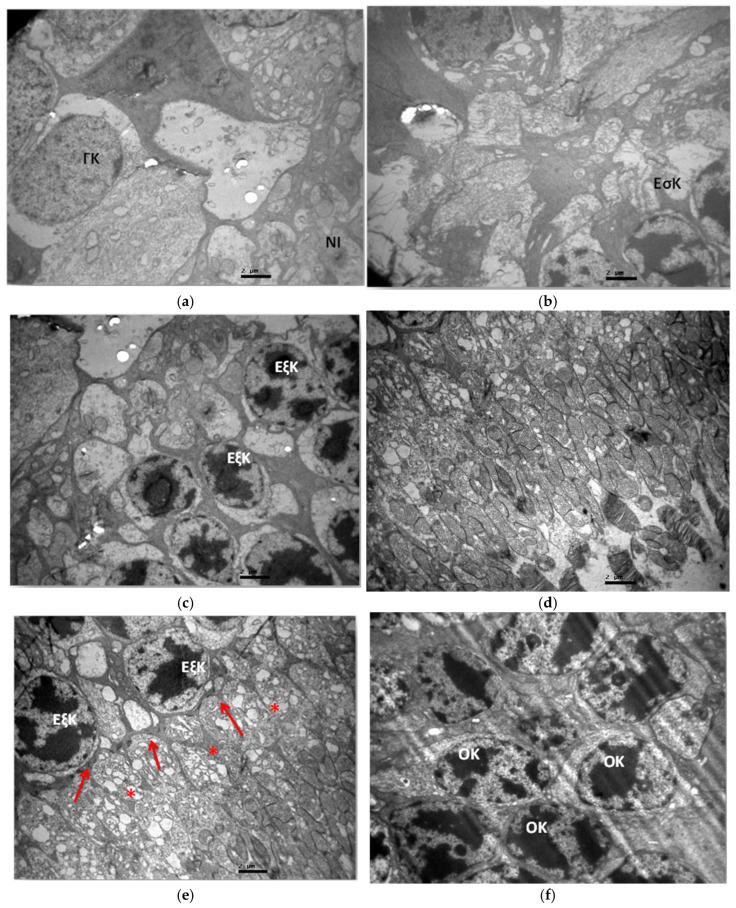
(**a**) Group C2. Ganglion cell layer (ΓΚ) and myelinated nerve fibers (NI) with absence of ultrastructural pathological lesions. ×6000. (**b**) Group C2. Inner granular layer (EσK) with absence of ultrastructural pathological lesions. ×6000. (**c**) Group C2. Outer granular layer (EξΚ) with absence of ultrastructural pathological lesions. ×6000. (**d**) Group C2. Photoreceptor layer with absence of ultrastructural pathological lesions. ×6000. (**e**) Group C2. Outer granular layer (EξK), inner part of photoreceptors (*) and outer limiting membrane (🡺) with absence of ultrastructural pathological lesions. ×6000. (**f**) Group C2. Outer granular layer, optic cells (OK) show absence of ultrastructural pathological lesions. ×6000.

The ultrastructural study of group C2 did not reveal any pathological lesions in any layer of the retina (Figure 13a–f).

#### 3.2.2. Group V2

**Figure 14:**
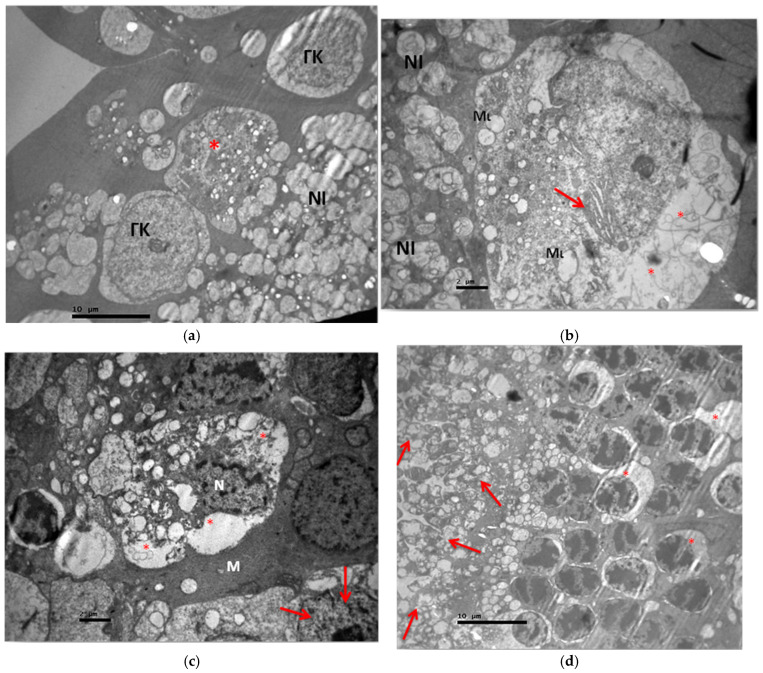

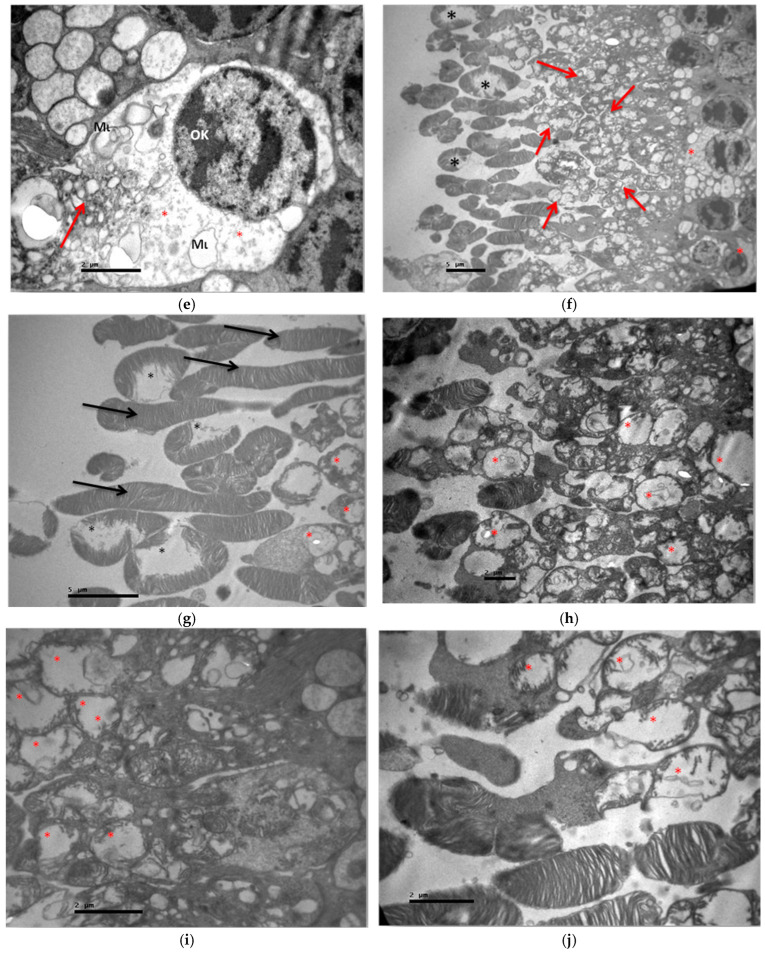
(**a**) Group V2. Ganglion cell in apoptosis (*) and presence of normal ganglion cells (ΓΚ) in the presence of normal nerve fibers (NI). ×2500. (**b**) Group V2. Ganglion cell with mitochondrial disorder (Μι), vacuolization in cytoplasm segment (*), RER enlargement (🡺) and presence of normal nerve fibers (NI) around it. ×5000. (**c**) Group V2. Bipolar cell in decay with vacuolar spaces (*) and nucleus (N) with nuclear membrane decay in the inner granular layer. Müller cell (M). Photoreceptor (🡺). ×6000. (**d**) Group V2. Rods and cones with vacuolated spaces and destruction of their normal architecture (🡺). External granular layer with local swelling of the cytoplasm (*). ×2500. (**e**) Group V2. Enlargement of the cytoplasm (*) and mitochondria (Mι) with disruption of their folds in the optic cells (OK) of the outer granular layer. Expansion of RER (🡺). ×12,000. (**f**) Group V2. Swelling and loss of normal mitochondrial architecture in the photoreceptor layer (🡺) Membrane disruption in the outer part of photoreceptors (*). Outer granular layer with local vacuolar spaces of the cytoplasm (*). ×3000. (**g**) Group V2. Outer part of the photoreceptors without strong lesions (🡺). Vacuolization in the outer part (*) to a lesser extent than in the inner part (*) of the photoreceptors. ×5000. (**h**) Group V2. Destruction and swelling of mitochondria (*) in the medial part of the photoreceptors. ×6000. (**i**) Group V2. Destruction and swelling of mitochondria (*) in the inner part of the photoreceptors. ×6000. (**j**) Group V2. Destruction of the inner parts of photoreceptors in the presence of cystoid mitochondria (*). Destruction and swelling of mitochondria. ×12,000.

The ultrastructural study of the V2 group revealed in the ganglion cell layer the presence of ganglion cells in the process of local apoptosis as well as the presence of normal ganglion cells (Figure 14a). Some ganglion cells had mitochondrial disorder, vacuolization in part of their cytoplasm, and enlargement of the rough endoplasmic reticulum (Figure 14a,b). In contrast, the myelinated nerve fibers in the nerve fiber layer did not show any pathological and morphological alterations (Figure 14a,b). In the inner granular layer, decaying bipolar cells with vacuolar spaces in their cytoplasm and disruption of their nuclear membrane were observed. Müller cells without morphological alterations were also identified (Figure 14c). In the outer granular layer, the optic cells showed enlargement of the cytoplasm and mitochondria with impaired folding, as well as enlargement of the rough endoplasmic reticulum (Figure 14d–f). Finally, in the layer of rods and cones, vacuolated spaces and destruction of the normal architecture of cones and rods were observed. The swelling and loss of normal mitochondrial architecture in the photoreceptor layer were particularly evident. The outer part of the photoreceptors did not show marked lesions compared to the inner part. The disruption of membranes in the outer part of the photoreceptors was observed to a lesser extent than in their inner part. The destruction of the inner parts of the photoreceptors in the presence of cystoid mitochondria was intense after their destruction and swelling. (Figure 14f–j). Ultrastructural lesions were observed in all retinal tissue samples.

#### 3.2.3. Group M2

**Figure 15:**
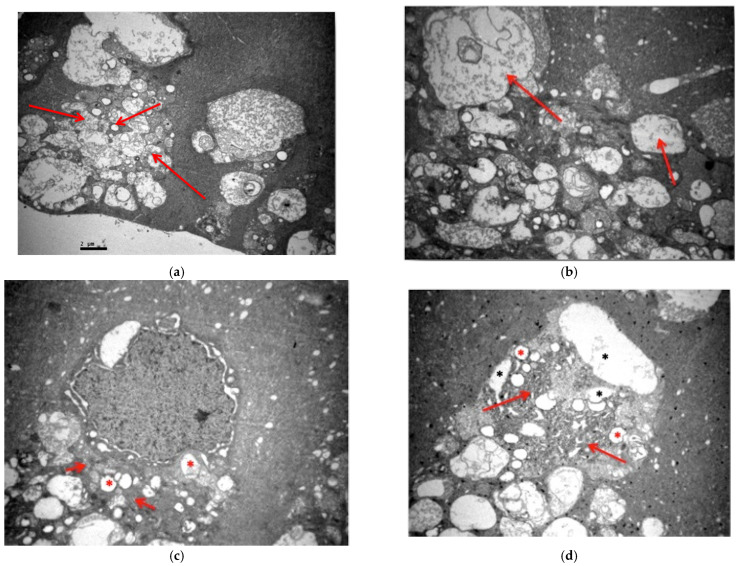

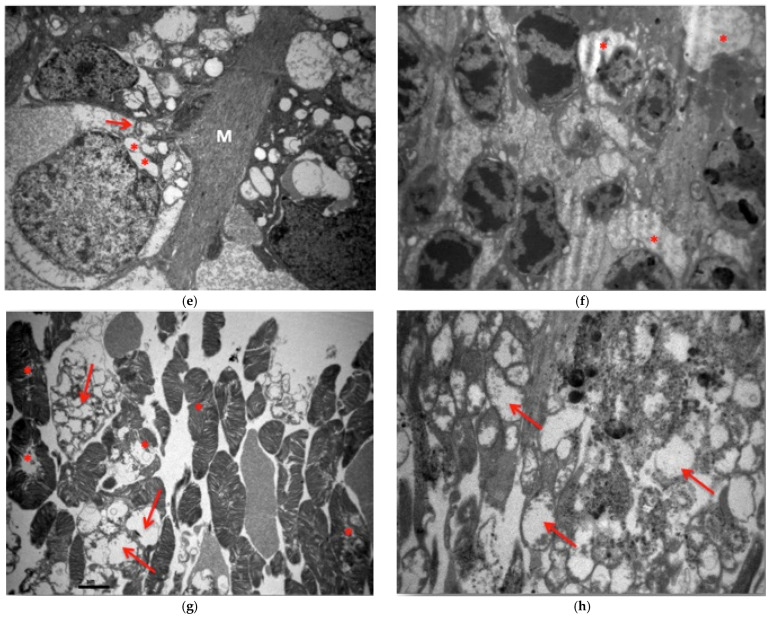
(**a**) Subgroup M2: Consolidation and fusion of unmyelinated nerve fibers with loss of their normal architecture (↑). ×6000. (**b**) Subgroup M2: Unmyelinated nerve fibers with local thinning of neurofibrils (↑). ×8000. (**c**) Subgroup M2: Ganglion cell with AED enlargement (↑) and destruction of the folds of certain mitochondria (*). ×8000. (**d**) Subgroup M2: Ganglion cell with AED enlargement (↑), destruction of the folds of some mitochondria (*) and presence of vacuolated spaces (*). ×8000. (**e**) Subgroup M2: Ganglion cell with AED enlargement (↑) and vacuolation of its cytoplasm (*). Müller cell (M). ×8000. (**f**) Subgroup M2: Outer granular layer with local vacuolation of optic cells (*). ×6000. (**g**) Subgroup M2: Layer of sticks and cones with loss of normal architecture of their outer part by disruption of their cell membrane (*), while in their inner part they present vacuoles and destruction in places of the cell membrane, as well as mitochondria with vacuolation and loss of membranes inside (↑). ×5000. (**h**) Subgroup M2: Layer of rods and cones with mitochondria showing vacuolation and destruction of membrane folds inside (↑). ×8000.

The ultrastructural study of the M2 group revealed consolidation and fusion of the myelinated nerve fibers with loss of their normal architecture in the nerve fiber layer (Figure 15a). Local thinning of their neurofibrils was also observed (Figure 15b). In the ganglion cell layer, ganglion cells showed enlargement of the coarse endoplasmic reticulum, local destruction of their normal mitochondrial folds, and vacuolation of their cytoplasm (Figure 15c–e). The Müller cells showed no pathological morphological alterations (Figure 15e). The outer granular layer showed local vacuolation of the optic cells (Figure 15f). Finally, the rod and cone layer showed a loss of the normal architecture of the outer part of the photoreceptors by disruption of their cell membrane, while the mitochondria were left enlarged and with loss of the inner membranous formations in the inner part of the rods and cones (Figure 15g,h). Ultramicroscopic lesions were observed in all retinal tissue samples.

#### 3.2.4. Group VM

**Figure 16:**
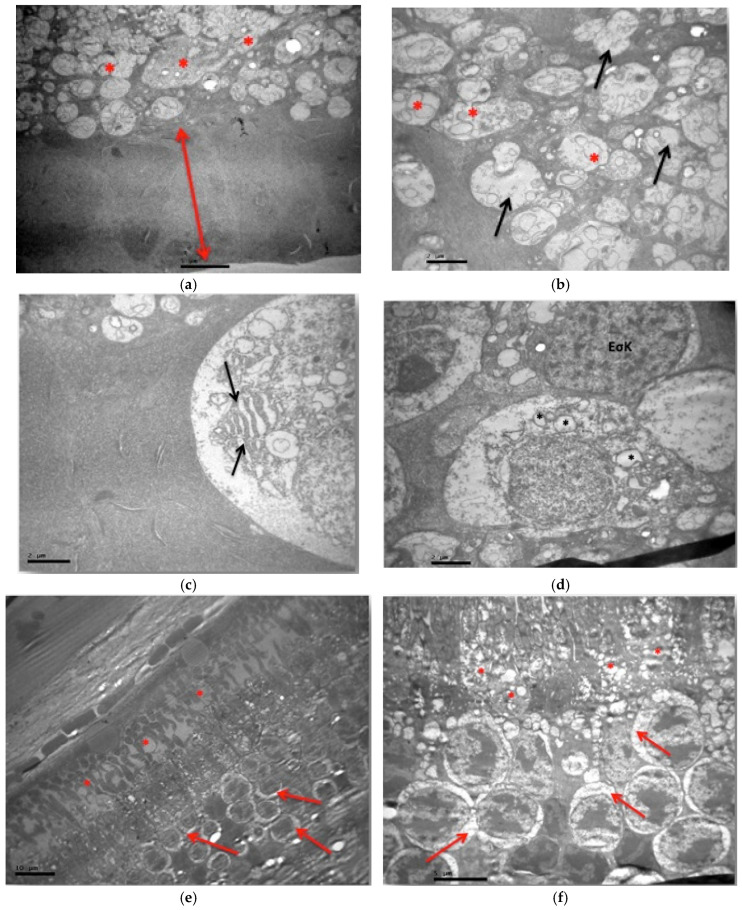

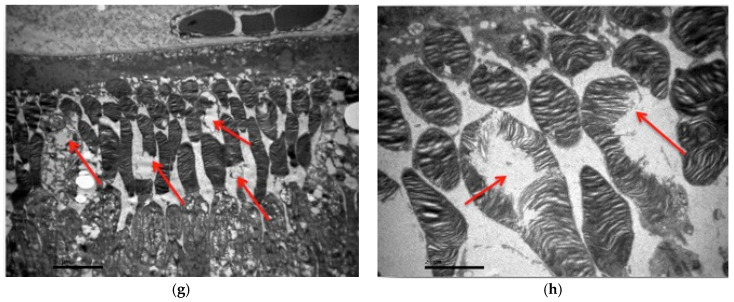
(**a**) Subgroup VM: Thickening of the inner aphoristic membrane (↕). Unmyelinaded nerve fibers in the nerve fiber layer (*). ×4000. (**b**) Subgroup VΜ: Mitochondria with damaged internal folds and presence of edema in the ganglion cell layer (*). Unmyelinated nerve fibers, with a small number of neurofibrils inside (↑). ×8000. (**c**) Subgroup VΜ: Ganglion cell with rough endoplasmic reticulum enlargement (↑). ×8000. (**d**) Subgroup VM: Mitochondria with damaged internal folds and presence of edema in the ganglion cell layer (*). Cells in the inner granular layer without lesions (EσK). ×8000. (**e**) Subgroup VM: Outer granular layer with vacuolated cytoplasm spaces (↑). Disturbance of the normal architecture of the outer part of the rods and cones (*). ×1500. (**f**) Subgroup VM: Sparsity of cytoplasm in the inner part of rods and cones (*). Outer granular layer with cytoplasm swelling (↑). Absence of an external hymen. ×4000. (**g**) Subgroup VM: Abnormal folds of the cell membrane of the outer part of the rods and cones. Segmentation and abnormal outgrowths of the cytoplasmic membrane with their local destruction in the photoreceptor layer (↑). ×4000. (**h**) Subgroup VM: Membrane disruption and local destruction of the outer fate of photoreceptors (↑). ×12,000.

The ultramicroscopic study of the VM subgroup revealed a thickening of the medial aphorism (Figure 16a). The unmyelinated nerve fibers had a small number of neurofibrils in sparsities (Figure 16b). In the ganglion cell layer, ganglion cells had mitochondria with damaged internal folds, edema in their cytoplasm, and enlargement of the rough endoplasmic reticulum (Figure 16c,d). The cells in the inner granular layer did not show any lesions. The outer granular layer presented vacuolar spaces in the cytoplasm of the optic cells, while the absence of the outer aphoristic membrane was characteristic (Figure 16d,e). In the layer of sticks and cones, disturbance of the normal architecture of the outer part was observed with abnormal folding and segmentation of the cell membrane, as well as local destruction of the outer fate of sticks and cones. Finally, in their inner part, thinning of the cytoplasm was observed (Figure 16e–h). Ultramicroscopic lesions were observed in all retinal tissue samples.

### 3.3. Statistical Analysis

Kruskal–Wallis analysis of variance demonstrated a statistically significant effect (*p* < 0.01) regarding the intensity of immunohistochemical staining in the detection of IL-6. The mild intensity (one cross) in all three samples of subgroup V1 made it statistically different from all other groups. Consequently, the single intravitreal injection of voriconazole was accompanied by a higher degree of IL-6 expression in the retinal tissue sections of rabbits compared to all other administrations of voriconazole and/or micafungin as well as to the control groups. The result of the Kruskal–Wallis analysis of variance was also statistically significant in the semiquantitative detection of TNF-a (*p* < 0.05). In contrast to what was observed for IL-6, mild immunohistochemical staining intensity was found in two of the three samples of the VM subgroup. In all other cases, this marker was not detected at all. In conclusion, TNF-a was only expressed (and to a mild extent) when voriconazole and micafungin were administered sequentially.

## 4. Discussion

Although rare, fungal endophthalmitis remains a significant clinical problem in ophthalmology due to the potential unfortunate consequences resulting from the severity of the infection. Additionally, fungal eye infections were very difficult to treat in the past due to limited treatment options available both systemically and intravitreal [55]. In recent years, there have been significant steps in the development of innovative antifungal agents, to achieve treatment regarding fungal endophthalmitis. New generation triazoles, such as voriconazole, posaconazole, and rabuconazole, represent the evolution in the class of triazoles and have been developed to address the increasing incidence of fungal infections and overcome the limitations of currently available agents [55,56,57,58,59]. Treatment with amphotericin B has been limited due to significant nephrotoxicity and relatively low intraocular penetration. Newer antifungal drugs such as fluconazole and voriconazole have broad-spectrum antifungal activity, significant bioavailability even with oral administration, and good ocular penetration. Voriconazole is a newer azole compound and the most promising as its therapeutic concentrations for most *Candida* and *Aspergillus* species are achieved in the vitreous body and can be used in infections resistant to fluconazole and other agents. Systemic therapy with either fluconazole (12 mg/kg loading dose, then 6–12 mg/kg daily) or voriconazole (6 mg/kg for two doses, then 4 mg/kg twice daily) is usually used for at least 4–6 weeks, with the final duration depending on the response observed using repeated eye examinations. Despite its significant systemic toxicity, amphotericin B may be used for the most serious infections when treatments with other agents have failed [58,59]. Although there is no standard treatment protocol, in cases where the macula is threatened and in cases of *Aspergillus* endophthalmitis, an intravitreal injection of an antifungal drug should be performed in addition to initiating systemic therapy. Voriconazole (100 μg/0.1 mL) or amphotericin B (5 μg/0.1 mL) is administered by intravitreal infusion. Voriconazole is considered safer than amphotericin B, but there is more experience with amphotericin B, which also has the advantage of having a longer half-life after intravitreal infusion. The need for repeated infusions depends on the response to treatment [58,59,60].

Early surgery with pars plana vitrectomy is recommended for cases of fungal endophthalmitis with significant vitreous involvement, in cases of suspected aspergillosis, or if there is no significant improvement with intravitreal therapy. Vitreous sampling at the time of vitrectomy can provide important material for culture and PCR to identify the pathogen to initiate appropriate treatment. The results of early vitrectomy in combination with systemic antifungals were favorable for cases of *Candida* and *Aspergillus* endophthalmitis. The half-life of antifungal agents administered directly to the vitreous body at the time of vitrectomy is reduced and repeated administration may be necessary if there is evidence of persistent infection [61,62]. As part of this thesis, we studied two antifungal agents that are the youngest representatives of the azole and echinocandins class. These are voriconazole and micafungin, respectively. Due to the limitations of the use of amphotericin B, the need to use alternative antifungal agents in safe doses and with the possibility of intravitreal administration is understood to limit possible side effects from systemic administration and to administer the smallest possible amount of medication. In addition, intravitreal administration is a short procedure that can be repeated many times to achieve the therapeutic goal. Of course, in order to prove the safety of using the two agents, it is necessary to study them at both microscopic and ultramicroscopic levels. In this study, the histological alterations caused by the maximum safe (based on the literature) doses of voriconazole and micafungin in intravitreal administration were examined to investigate the safety of their possible use in humans and their possible causal relationship with the often-poor outcome of cases with fungal endophthalmitis.

However, few studies have been published on the histological effects of the two agents on the retina after intravitreal administration, and there is a lack of studies using an electron microscope to highlight the ultramicroscopic lesions caused by these agents in the retina. Most studies refer to the clinical features of drug use, especially in cases where an intraocular fungal infection has occurred. Most experimental protocols study the induction of fungal intraocular infection and the therapeutic response to different doses of antifungal agents. There is also a lack of data on the histological effects of drugs on the retina without prior fungal infection, except the study by Harrison and colleagues, where a histological study was conducted by applying eosin–hematoxylin staining to rabbit eyes treated with both voriconazole and micafungin. However, histological changes were only observed in the eyes where the medicine was given in combination with fungal infection, and no changes were observed in the eye injected alone. In terms of dosages, intravitreal infusion of micafungin at a dose of 0.06 mL containing 15 μg micafungin and voriconazole infusion at a dose of 0.06 mL containing 150 μg voriconazole had been performed [48].

More specifically, Harrison, Glickman, and colleagues compared the intravitreal administration of amphotericin B, voriconazole, and micafungin in rabbit eyes with the study of electroretinography and histological alterations with the application of eosin–hematoxylin staining. They concluded that amphotericin B and micafungin are equally effective at maintaining retinal function in the first 72 h after administration; however, micafungin is less toxic. On the other hand, they concluded that voriconazole has a disadvantage in maintaining retinal function compared to the other two substances, requires higher concentrations, and is considered more toxic than micafungin. Additionally, this protocol refers to the administration of only one intravitreal injection and not multiple [48]. However, our results contradict Harrison’s. Micafungin in the single injection protocol did not cause microscopic lesions, in contrast to two infusions, which caused alterations in the ganglion cell layer, while ultra-microscopic lesions were found in almost all retinal layers in all samples with both micafungin infusions, and indeed with the lesions in the double infusion being more extensive and intense than with one micafungin infusion. Therefore, micafungin is toxic to the retina and, based on the extent and intensity of findings, appears to be more toxic than voriconazole.

In addition, the study by Gao and colleagues in rat eyes showed that intravitreal administration of voriconazole to achieve an intravitreal concentration of 5 to 25 μg/mL does not cause electroretinal or histological changes in the retina, while an intravitreal concentration between 50 μg/mL and 500 μg/mL causes small foci of retinal necrosis, with disorganization in particular of the photoreceptor layer and the inner granular layer, as well as photoreceptor degeneration. In contrast, the ganglion cell layer remained intact. At intravitreal concentrations above 500 μg/mL, voriconazole caused more focal necrotic regions in the retina with more apparent photoreceptor degeneration and disorganization of the photoreceptor layer and the inner granular layer. In fact, focal retinal detachment was observed in these necrotic areas. Remarkably, inflammatory cells were also observed in these focal areas of the retina in the presence of choroid congestion. The parameters statistically compared were the b- and a-wave in the ERG, while no statistical analysis was performed on the histological findings. The intravitreal concentration of 25 μg/mL in rat eyes corresponds to a dose of 100 μg in the human eye and does not cause long-term lesions. Therefore, according to Gao, voriconazole is considered safer to use than amphotericin B in intravitreal infusion and suggests the intravitreal concentration of voriconazole up to 25 mg/mL is safe [51]. However, this protocol was implemented by administering only one intravitreal injection and not multiple injections [44]. In our protocol, we further studied the ultramicroscopic lesions caused by the apparently safe intravitreal concentration of voriconazole of 25 μg/mL, in both one and two intravitreal injections, and observed alterations in all samples.

In fact, with one injection, ultramicroscopic lesions were found in the nerve fiber layer, the ganglion cell layer, and the outer granular layer, while with the two injections more intense lesions were found in the aforementioned layers as well as additional lesions in both the inner granular layer and the rod and cone layer. Of course, the application of eosin–hematoxylin staining did not reveal any alterations, confirming the effects of Gao and the characterization of this voriconazole concentration as safe based only on the microscopic lesions it causes. Additionally, the ERG offers a general indication of retinal function and may not be able to detect (multi)focal histopathological lesions, to the extent that they have not expanded sufficiently to cause a significant impairment of the retina’s overall function [52]. Therefore, our research reveals ultramicroscopic alterations that contradict the picture of the safe profile of the intravitreal concentration of voriconazole at 25 μg/mL. Further, unlike Gao’s findings, where inflammatory cells were found in the focal necrotic regions of the retina when high doses of voriconazole were administered, in our protocol with the single infusion of voriconazole, the immunohistochemical marker IL-6 was detected as mildly positive in all samples in the presence of lymphocytes. However, the role of IL-6 in causing retinal damage after intravitreal voriconazole injection is not clear, as no lymphocytes or other inflammatory cells were found during the observation of the samples under the electron microscope, while paradoxically the staining was negative in the protocol of the two infusions.

As for micafungin, according to Paris and colleagues, intravitreal administration of micafungin at a dose of 150 μg to rabbit buds does not cause alterations in the ERG [36]. However, the ERG was the only means of detecting retinal damage, as no histological analysis was performed. While according to Kapur, intravitreal administration of micafungin to rabbit eyes at a dose up to 0.025 mg/0.1 mL did not cause histopathological lesions, nor alterations in the ERG, suggesting this dose as a safe non-toxic starting dose with sufficient antimicrobial activity in experimental studies and therefore for future use in humans for the treatment of fungal endophthalmitis [38]. Our findings are consistent with those of Kapur for the same dose of micafungin, 0.025 mg/0.1mL, and eosin–hematoxylin staining revealed no morphological alterations in the retina after an intravitreal infusion, making it an apparently safe dose for intravitreal administration. However, after two intravitreal injections of micafungin, the eosin–hematoxylin stain revealed morphological alterations in the ganglion cell layer, in particular shrinkage of the ganglion cell cytoplasm. Additionally, the ultramicroscopic lesions observed in almost all retinal layers with both one and two intravitreal injections overturn the apparently safe profile of micafungin at a dose of 0.025 mg in intravitreal infusion.

However, many studies have been published on the systemic and topical administration of both voriconazole and micafungin. Their therapeutic adequacy and safety of use have been studied in many clinical cases and experimental protocols, mainly through intravenous and oral administration and to a lesser extent through local administration in the form of an instillation solution on the cornea and into the anterior chamber. In particular, voriconazole has been widely used in the treatment of ocular fungal infections through local, oral, and intraocular routes of administration. Many studies have been conducted to study its mode of action, its activity against different species of fungi, its bioavailability, and its pharmacokinetics, as well as its toxicity and associated adverse reactions. Due to the complex and highly sensitive metabolism of voriconazole, many challenges remain unresolved regarding the adequacy of dosing regimens. Thus, under- and overdosing of voriconazole often occur, resulting in therapeutic deficiency and the development of side effects [63,64]. While further studies are apparently needed to determine the appropriate level of voriconazole for use in humans, this agent has been used in selected cases alone or with another intravenous antifungal drug (caspofungin), with no evidence of apparent toxicity [60]. In one study, postoperative fungal endophthalmitis was successfully treated using intravitreal and anterior voriconazole infusion as the sole therapeutic agent without combination therapy [65]. However, our protocol did not involve causing a fungal infection.

## 5. Conclusions

The present research study is characterized by novel and original findings, being the first to evaluate the ultramicroscopic lesions as well as the immunohistochemical effects of intravitreal voriconazole and micafungin—separately, in repeated doses, and in combination—at doses regarded as safe based on earlier experimental protocols. While past research primarily relied on histological assessment using hematoxylin–eosin staining, this investigation fills a critical gap in the literature by incorporating transmission electron microscopy and immunohistochemical analysis of IL-6 and TNF-a, thereby uncovering subclinical changes in retinal integrity.

In summary, the conclusions of this research paper are the following: Voriconazole did not cause visible histological alterations on light microscopy in either single or repeated administrations. However, electron microscopy revealed clear ultrastructural damage and hyper-microscopic lesions, which indicates its possible toxic effect on the retina, despite its apparent safety.

In fact, with one injection, lesions were found in the nerve fiber layer, the ganglion cell layer, and the outer granular layer, while with the two injections, more intense lesions were found in the aforementioned layers, as well as additional lesions in both the inner granular layer and the rod and cone layer. These findings suggest that voriconazole exerts a dose-dependent toxic effect on retinal cells, despite its otherwise acceptable safety profile based on routine histology. Therefore, voriconazole is more toxic in a multidose protocol. The immunohistochemical marker TNF-a does not appear to play a pathogenetic role in the retinal alterations caused by voriconazole, as it was found to be negative in all samples with the single and double voriconazole infusion, even though cells in apoptosis were found in the samples with the double voriconazole infusion. Although IL-6 tested positive in samples with single voriconazole infusion, it tested negative in both voriconazole injections and micafungin combined infusions, leading to conflicting conclusions regarding the role of IL-6-mediated inflammation in voriconazole causing retinal lesions.

Micafungin in a single infusion protocol did not cause microscopic lesions, unlike its two infusions that caused alterations in the ganglion cell layer. However, ultramicroscopic lesions in almost all layers of the retina were found in all samples with both micafungin injections, and even with the lesions in the double infusion being more extensive and intense than with the single micafungin infusion. Therefore, micafungin is toxic to the retina and, based on the extent and intensity of these findings, appears to be more toxic than voriconazole. The immunohistochemical marker TNF-a does not appear to play a pathogenetic role in the lesions caused by micafungin in the retina, as it was found to be negative in all samples with the single and double micafungin injection, indicating that apoptosis does not belong to the mechanisms of retinal damage. The immunohistochemical marker IL-6 also does not appear to play a pathogenetic role in micafungin’s retinal lesions, as it tested negative in all samples with single and double micafungin infusion, indicating that IL-6-mediated inflammation does not contribute to micafungin’s retinal lesions. Finally, in the combined administration of voriconazole and micafungin, both microscopic and ultramicroscopic lesions were found in almost all layers and especially in the layer of ganglion cells and photoreceptors, indicating the toxic effect on the retina of the combination administration regimen. In fact, ultramicroscopic lesions were enhanced by the detection of microscopic lesions in the photoreceptor layer, as well as by the positive expression of the immunohistochemical marker TNF-a in two of the three eyes in the ganglion cell layer. Therefore, apoptosis appears to belong to the mechanisms of retinal damage caused by the combined administration of voriconazole and micafungin. In contrast, IL-6-mediated inflammation does not contribute to retinal lesions caused by the combined administration of the two agents.

These findings raise significant considerations for clinical ophthalmology, particularly in the context of intravitreal antifungal therapy for fungal endophthalmitis. Both voriconazole and micafungin, agents commonly used in ophthalmology because of their antifungal efficacy, can induce subclinical retinal toxicity when administered intravitreally, particularly in multiple doses or in combination. Voriconazole’s and micafungin’s impact on the retina may not be detectable through routine histology or standard ophthalmic examination but could contribute to subtle retinal dysfunction or long-term retinal damage. These findings support the need for more rigorous monitoring protocols in patients receiving voriconazole and micafungin, mainly those requiring repeated dosing. Alternative dosing strategies, protective adjuncts, or modified formulations, in order to minimize retinal exposure by maintaining simultaneous antifungal efficacy, should also be explored.

## Figures and Tables

**Table 1:** Animal Randomization Tool groups assortment, with a number ID assigned to each rabbit from 1 to 9; female (XX), male (XY).

GROUPS	V2	M2	VM	C
NUMBER ASSIGNED	7, 4, 3	1, 9, 2	6, 5, 8	1, 2, 3, 4, 5, 6, 7, 8, 9
GENDER	XX, XX, XX	XY, XY, XX	XY, XY, XX	XY, XX, XX, XX, XY, XY, XX, XX, XY

## Data Availability

The original contributions presented in this study are included in the article. Further inquiries can be directed to the corresponding authors.
